# Small bowel mucormycosis: An unexpected case in a penetrating trauma survivor

**DOI:** 10.1016/j.ijscr.2023.109071

**Published:** 2023-11-19

**Authors:** Malini Bhana, Naadiyah Laher, Nathan George McGrath, Maeyane Stephens Moeng

**Affiliations:** a Division of Trauma, Department of General Surgery, Charlotte Maxeke Johannesburg Academic Hospital, 5 Jubilee Street, Parktown, Johannesburg, South Africa; b Division of Anatomical Pathology, National Health Laboratory Service, Charlotte Maxeke Johannesburg Academic Hospital, 5 Jubilee Street, Parktown, Johannesburg, South Africa

**Keywords:** Penetrating, Trauma, Small bowel, Mucormycosis, Outcomes

## Abstract

**Introduction:**

Small bowel mucormycosis is a rare entity with few reports in the literature. Mortality rates secondary to necrosis and perforation remain above 85 %, with an increase in populations at risk noted.

**Presentation of case:**

This is a case report of a survivor of penetrating trauma who sustained small bowel injuries and was managed with damage control surgery. He required relook laparotomies due to extensive contamination and subsequently developed progressive ischaemia and necrosis of areas of his small bowel – histology confirming mucormycosis. There were no apparent risk factors noted in this case. Early addition of Amphotericin B and prompt surgical management resulted in a positive outcome. The patient was discharged from the hospital successfully. No further complications were noted post-discharge.

**Discussion:**

Small bowel mucormycosis can be a challenging diagnosis and requires a high index of suspicion. The lack of traditional risk factors should not deter a surgeon from considering this diagnosis in trauma patients as the micro-invasive properties of this organism can result in unexpected gastrointestinal ischaemia. Favourable outcomes are associated with prompt surgical debridement, histopathological diagnosis, and appropriate antifungal therapy.

**Conclusion:**

Gastrointestinal Mucormycosis is a diagnosis that should be considered in trauma patients with unusual patterns of ischaemia. Prompt therapy can result in positive outcomes.

## Highlights

• Small bowel mucormycosis is rare but can occur in trauma patients.
• A high index of suspicion and identification of this rare cause is essential.
• Prompt surgical debridement and Amphotericin is important for good patient outcomes.

## 1 Introduction

Gastrointestinal (GIT) mucormycosis is an uncommon, aggressive, angioinvasive infection, largely affecting the stomach – with very few reported cases of jejunal involvement [1]. Mortality remains high at up to 85 % with gastrointestinal involvement, largely due to ischaemia, necrosis and subsequent bowel perforation [2]. Globally, an increasing trend in populations at risk has been observed. However, an interesting finding in the literature is that in its gastrointestinal(GIT) manifestations – it frequently occurs in patients without the typical risk factors - highlighting the need for clinicians to maintain a high index of suspicion [1]. We present a case of jejunal mucormycosis post penetrating trauma where early recognition and intervention allowed for improved patient outcomes in this invasive fungal infection.

## 2 Presentation of case

A 29-year old man presented to our Level 1 Trauma unit in Johannesburg following a penetrating stab injury to the abdomen. He sustained a 10 cm laceration over his left lower back with subsequent bowel evisceration. The bowel was noted to be dusky on arrival, with associated perforation. He came in hypotensive with a blood pressure of 75/55 mmHg and a pulse rate of 109. He was resuscitated in our emergency department as per ATLS® [3] principles and planned for a laparotomy. A massive transfusion protocol was activated as his blood gas on arrival revealed a metabolic acidosis with a pH of 7.19, bicarbonate (HCO3) of 15, lactate of 12.8 mmol/l and Hb of 6.6 g/dl. He was taken to theatre within hours of arrival, and a midline laparotomy was performed where 1000 ml haemoperitoneum was found with an American Association for the Surgery of Trauma (AAST) Grade III small bowel and a left Zone 2 retroperitoneal haematoma as well as an associated AAST Grade IV left ureteric injury.

The small bowel perforations were debrided and primarily repaired. The left ureter was managed, and bleeding from the lumbar vessels was controlled by means of haemostatic sutures. He received a massive transfusion in the form of four units of packed red cells, four units of fresh frozen plasma and one mega-unit of platelets intra-operatively. Due to the patient's ongoing haemodynamic and metabolic instability combined with his coagulopathy, swabs were packed into the retroperitoneal bed of the injury site, and temporary abdominal closure was achieved by means of a vacuum-assisted dressing. He was then transferred for further resuscitation in the Intensive care unit (ICU), and a subsequent relook laparotomy was planned for definitive surgery as part of damage control principles [3].

His goal-directed resuscitation continued in the ICU, where he was weaned off low-dose inotropes (adrenalin at 0.05 μg/kg/h) with an improving metabolic acidosis and lactate of 1.5 on Day 1 post-surgery. As per unit protocols, only preoperative prophylactic antibiotics were given. He was noted to have an acute kidney injury (KDIGO Stage 1) without any acute indications for dialysis. Electrolyte imbalances were corrected, and he was then started on enteric feeds via his nasogastric tube.

At his planned relook, small bowel repairs remained intact with ongoing contamination noted in the peritoneal cavity due to a missed posterior gastric wall injury (AAST) Grade II. Given the contamination, he was started on empiric broad-spectrum antibiotics (Amoxycillin-Clavulanic acid), an antifungal (Fluconazole), and planned for a further relook and washout in 48 h. He required an additional unit of blood intraoperatively, and his acute kidney injury remained unchanged. His antibiotics were subsequently escalated to empiric Imipenem following positive gram-negative bacillus blood cultures as per unit antibiogram and microbiology recommendation.

**Fig. 1:**
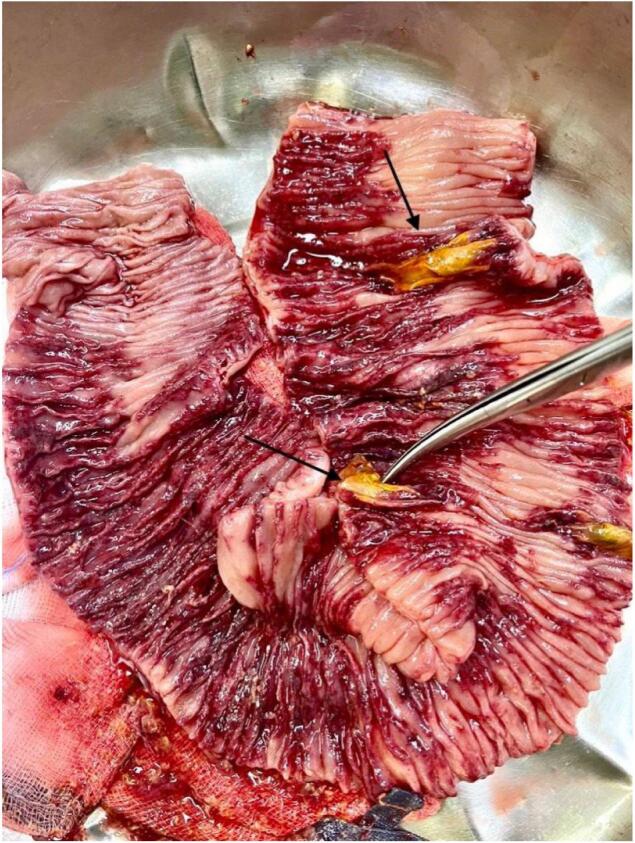
Small bowel with areas of necrosis and perforation found intra-operatively.

**Fig. 2:**
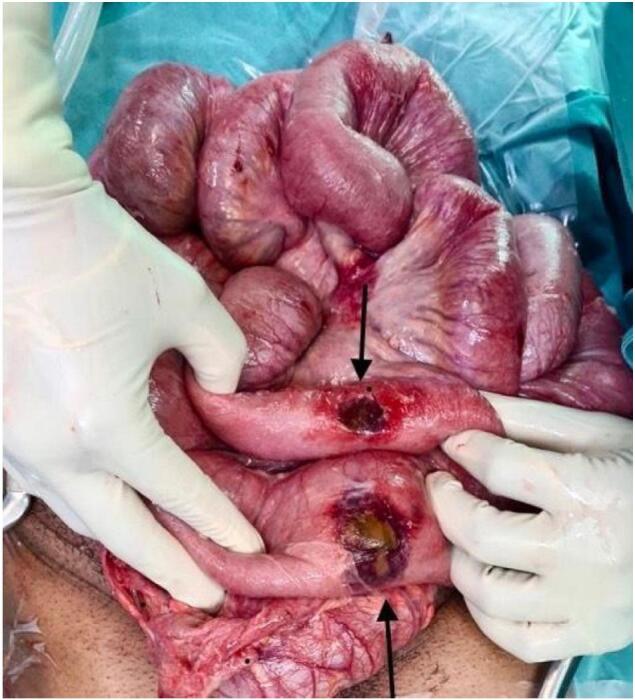
Small bowel with areas of necrosis and perforation found intra-operatively.

**Fig. 3:**
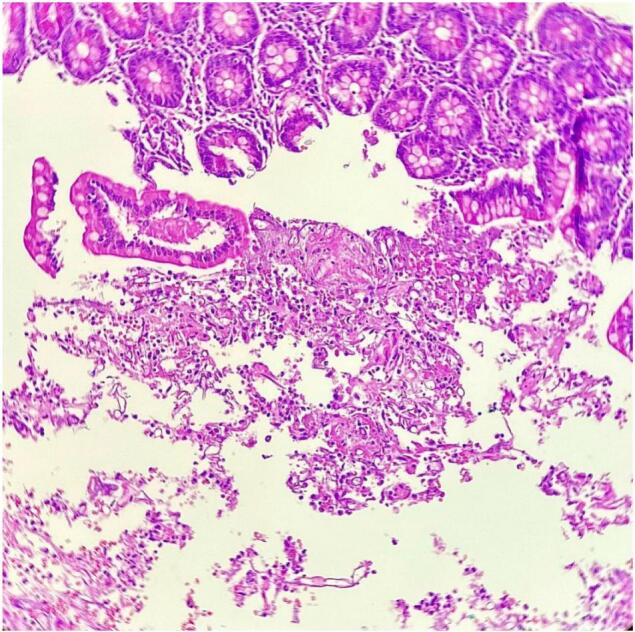
A higher power (10× magnification) view of sloughed off epithelium, fibr finosuppurative exudate and fungal micro-organisms with broad non-septate hyphae and right angle branching.

**Fig. 4:**
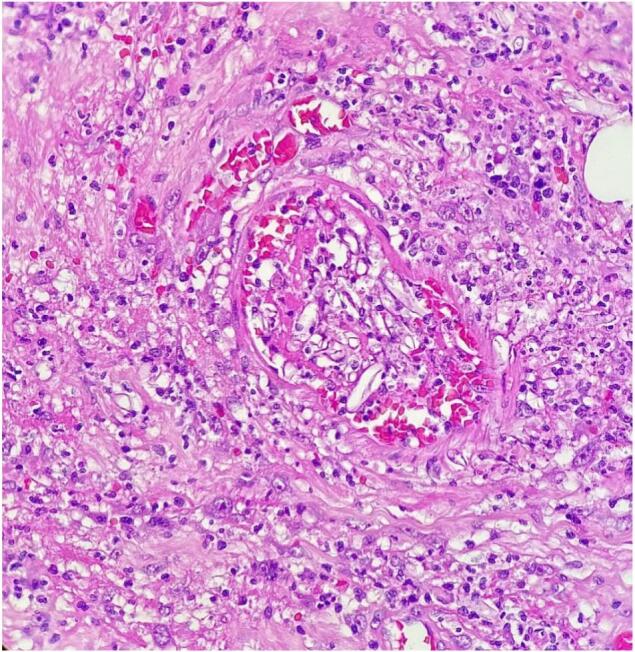
High power (40×) magnification of the angioinvasive fungal micro-organisms invading vessel walls.

Suspicious lesions unrelated to his initial injuries were noted on the small bowel at his further relook (done due to extensive contamination at prior relook). The patchy features then progressed at follow-up surgery, where resection of the affected segment of the bowel was carried out (Fig. 1, Fig. 2). Antifungal therapy was escalated to Amphotericin B with a good clinical outcome at this stage. Histology of the jejunal resection confirmed mucormycosis, with thickened areas of bowel wall noted macroscopically. Microscopically, there was extensive mucosal necrosis and suppurative inflammation (Fig. 3). Areas of necrotizing granulomatous inflammation were observed, with numerous fungal micro-organisms. These fungal micro-organisms exhibit broad, pauci-septate hyphae that branch at 90-degree angles with angioinvasion noted (Fig. 4).

The patient completed a 21-day course of Amphotericin B as recommended by the infectious disease specialists, with normal renal function on completion. He was discharged from the hospital with normal outpatient follow-up, revealing healthy weight gain and regular bowel habits.

## 3 Discussion

The most common site of GIT mucormycosis is the stomach, followed by the colon, with jejunal involvement being very rare in the literature [1]. Mortality is usually high as a result of bowel perforation or GIT bleeding [2]. Another significant observation in GIT mucormycosis is its common incidence in a host without classical risk factors. In adults, its progression has largely been associated with an immunocompromised state, most commonly with diabetes mellitus and HIV [4].

In immunocompetent hosts, postulated risk factors include burns and nosocomial infections associated with cutaneous mucormycosis. Trauma is another postulated factor, with a study in India showing an up to 22 % association with cutaneous mucormycosis [5]. In this patient, no readily identifiable risk factors were found; he was not on any immunosuppressive medication and workup for diabetes mellitus, HIV, and occult malignancy were all negative. Possible contributors to an immunocompromised state would be his significant injuries sustained by trauma and massive blood transfusions required on top of multiple relook laparotomies.

The pathologic hallmark of mucormycosis is infarction of host tissue resulting from angioinvasion by hyphae. This gives rise to necrotic ulcers with resultant acute abdominal pain, hematemesis, perforation and peritonitis [6]. Given the rapid progression, the prognosis is poor. Only 25 % of cases are diagnosed antemortem [7]. The need for histopathological diagnosis hampers early diagnosis to improve outcomes. However, one needs to identify at-risk patients or suspicious surgical findings to have the insight to request specific histopathological investigations for mucormycosis. Unfortunately, due to its rarity, it is not commonly looked for. Confounding factors may further hamper diagnosis, as in this patient where contributory factors like multiple relooks and possible hypoperfusion secondary inotropic support resulted in a low index of suspicion for mucormycosis.

Key strategies to avoid this dismal prognosis are early diagnosis, prompt surgical management, and antifungal therapy, as was the case in our patient. Recommendations at large are not specific, however, and there is no recognized optimal course of therapy. The initiation of a polyene therapy (usually Amphotericin B) within five days of diagnosis has been associated with survival improvement [8]. While novel azoles like Isavuconazole are now considered for mucormycosis – the azoles are, in general, not an effective therapy and, as in this patient, need to be escalated to Amphotericin B. Poor tissue penetration as a result of thrombosis and necrosis caused by mucormycosis however poses an added problem.

Surgical debridement of infected tissue thus appears to be a critical component to complete eradication [2], and given the high mortality attributable to GIT mucormycosis, aggressive surgical management is vital and timely resection of infected tissue was indeed critical in our patient and likely contributed to his favourable outcome. Guidelines as to therapy duration and dose are, however, scanty. Expert opinion is that antifungal therapy should be continued until symptom resolution or resolution of underlying risk factors [1]. This may pose a problem in populations receiving immunosuppressive therapy, as is the case in South Africa, and in these cases, secondary fungal prophylaxis should possibly be continued [9]. In our case, antifungal therapy was discontinued upon resolution of symptoms, and as no risk factors were readily identifiable, secondary prophylaxis was not initiated.

## 4 Conclusion

Given the global increase in at-risk patients with the steadily rising epidemic of obesity and diabetes mellitus and our population with HIV and tuberculosis, there is a dire need for improved diagnosis and management of mucormycosis [10]. At this stage, however, it remains a clinical diagnosis with an emphasis on clinicians to keep a high index of suspicion and implement timeously the appropriate diagnostic evaluation and management if patient outcomes are to be improved. GIT, particularly jejunal mucormycosis, is a rare yet debilitating infection not commonly seen in trauma. In our case, early surgical debridement and prompt antifungal therapy are believed to be responsible for this admirable outcome.

## 5 Method

The work has been reported in line with the SCARE criteria [11].

## Consent

Written consent has been obtained from the patient.

## Ethical approval

Ethics Approval was obtained ON 06/09/2023. Protocol No M230972.

## Funding

No funding was required for this research.

## Author contribution

Malini Bhana – writing of paper.

Naadiyah Laher – writing of paper.

Maeyane Moeng - organized the manuscript and revised the paper.

Nathan George McGrath – contributed the histology and slides.

## Guarantor

Prof MS Moeng.

## Research registration number

N/A.

## Conflict of interest statement

The authors did not require any funding and have no conflicts of interest to declare.
